# Distinct roles for the RNA-binding protein Staufen1 in prostate cancer

**DOI:** 10.1186/s12885-021-07844-2

**Published:** 2021-02-04

**Authors:** Kristen A. Marcellus, Tara E. Crawford Parks, Shekoufeh Almasi, Bernard J. Jasmin

**Affiliations:** 1 Department of Cellular and Molecular Medicine, Faculty of Medicine, University of Ottawa, 451 Smyth Road, Ottawa, Ontario K1H8M5 Canada; 2 The Eric J. Poulin Centre for Neuromuscular Diseases, Ottawa, Ontario Canada

**Keywords:** Prostate cancer, Staufen1, RNA-binding proteins, Invasion, Migration, Proliferation

## Abstract

**Background:**

Prostate cancer is one of the most common malignant cancers with the second highest global rate of mortality in men. During the early stages of disease progression, tumour growth is local and androgen-dependent. Despite treatment, a large percentage of patients develop androgen-independent prostate cancer, which often results in metastases, a leading cause of mortality in these patients. Our previous work on the RNA-binding protein Staufen1 demonstrated its novel role in cancer biology, and in particular rhabdomyosarcoma tumorigenesis. To build upon this work, we have focused on the role of Staufen1 in other forms of cancer and describe here the novel and differential roles of Staufen1 in prostate cancer.

**Methods:**

Using a cell-based approach, three independent prostate cancer cell lines with different characteristics were used to evaluate the expression of Staufen1 in human prostate cancer relative to control prostate cells. The functional impact of Staufen1 on several key oncogenic features of prostate cancer cells including proliferation, apoptosis, migration and invasion were systematically investigated.

**Results:**

We show that Staufen1 levels are increased in all human prostate cancer cells examined in comparison to normal prostate epithelial cells. Furthermore, Staufen1 differentially regulates growth, migration, and invasion in the various prostate cancer cells assessed. In LNCaP prostate cancer cells, Staufen1 regulates cell proliferation through mTOR activation. Conversely, Staufen1 regulates migration and invasion of the highly invasive, bone metastatic-derived, PC3 prostate cells via the activation of focal adhesion kinase.

**Conclusions:**

Collectively, these results show that Staufen1 has a direct impact in prostate cancer development and further demonstrate that its functions vary amongst the prostate cancer cell types. Accordingly, Staufen1 represents a novel target for the development of much-needed therapeutic strategies for prostate cancer.

**Supplementary Information:**

The online version contains supplementary material available at 10.1186/s12885-021-07844-2.

## Background

Prostate cancer (PC) is one of the most common malignant cancers with the second highest rate of mortality in men worldwide [1–4]. Despite improved scientific knowledge surrounding the underlying molecular mechanisms and risk factors associated with PC, prognosis remains poor for advanced PC [5]. The high mortality rate associated with PC is a result of aggressive migration and invasion of PC cells [6]. PC develops from prostatic intraepithelial neoplasia (PIN), by means of progressive changes in normal prostatic epithelial cells to premalignant lesions [7, 8]. PC is often detected by a combination of testing for prostate-specific antigen, where levels > 4 ng/ml indicate an increased risk of prostate cancer, and tissue biopsy [5, 9].

Initially, patients diagnosed during the early stages of PC, have an androgen-dependent tumour that is confined to the prostate capsule. Primary treatment involves radiation therapy or prostatectomy [4, 10, 11]. However, if the cancer spreads outside of the prostate, androgen deprivation therapy is required [10–12]. Unfortunately, androgen ablation therapy eventually fails and the majority of patients progress to castration-resistant prostate cancer (CRPC), in a median of 12 to 18 months after androgen deprivation therapy [10, 12]. Given the poor prognosis of advanced PC, there is a strong need for additional research focused on the molecular mechanisms underlying the progression of CRPC.

A key hallmark of CRPC is the ability to invade tissues and establish metastatic sites [13]. As metastatic disease is the leading cause of death in cancer patients, investigation into the process of tumour invasion and migration is essential for identifying new therapeutic targets [13]. Metastasis is the formation of a secondary tumour at a distant site from the primary lesions [14, 15]. The process of metastasis is multifaceted. It begins with a cancer cell breaking away from the primary tumour and infiltrating the circulatory system, followed by invasion of a different tissue and growth at that secondary site [14, 16]. The cellular processes implicated in metastasis are regulated by receptors such as integrins and intracellular proteins that control adhesion to the surrounding extracellular matrix, including focal adhesion kinase (FAK) [17–27]. Another aspect of metastasis is the loss of growth regulation, leading to uncontrolled proliferation, which is regulated by several signaling pathways including PI3K/AKT and mTOR [17, 28–36].

Recently, we described the involvement of the RNA-binding protein (RBP) Staufen1 in Rhabdomyosarcoma (RMS) tumorigenesis [37]. Our findings showed that Staufen1 is upregulated in RMS and contributes to the pathogenesis by controlling proliferation, migration, and invasion of RMS cells [37]. Historically, Staufen1 is well known as a multi-functional, double-stranded RBP involved in several key aspects of RNA metabolism including mRNA localization, transport [38, 39], stability [40–42], translation [37, 43–47], nuclear export [48, 49], the cell cycle [50] and alternative splicing [46, 49, 51]. These functions are all key for cell physiology and homeostasis, and highlight the fact that misregulation of Staufen1 likely has major detrimental impacts on cell function.

Currently, there is a paucity of information on the impact of Staufen1 in cancer biology [37, 50, 52–58]. Yet, its implication in several key RNA processes in a variety of cell types indicates that it likely plays pivotal roles in cancerous cells. In this context, its potential function in prostate cancer is unknown. Since the molecular mechanisms underlying metastatic cancer include cell motility, invasion and proliferation of cells, we investigated in the present study how Staufen1 regulates these functions in prostate cancer cells. Here, we report that Staufen1 is markedly increased in PC. Furthermore, and importantly, Staufen1 plays differential roles in prostate cancer cells by regulating either cell growth or migration/invasion. Our study highlights for the first time, therefore, the oncogenic role of Staufen1 in PC as well as its potential as a novel biomarker and target for much-needed cancer therapeutics.

## Methods

### Constructs and antibodies

The constructs used were pLKO.1-TRC cloning vector, a gift from David Root (Addgene #10878), pLKO.1-TRC-shStau1 (Clone ID: TRCN0000102306, Clone ID: TRCN0000102308 and Clone ID: TRCN0000102309) (GE Healthcare Life Sciences, Ontario, Canada), pcDH-CMV-MCS-EF1-copGFP and pcDH-Stau1-HA (as previously described [49]) pMD2.G (Addgene #12259) and psPAX2 (Addgene #12260) were gifts from Didier Trono.

The antibodies used were anti-Staufen1 (ab73478, Abcam, Ontario, Canada), anti-phospho (Ser2448)-mTOR (#2971, Cell Signaling Technology, Danvers, MA, USA), anti-mTOR (#2983, Cell Signaling Technology, Danvers, MA, USA), anti-phospho (Try576/577)-FAK (#3281, Cell Signaling Technology, Danvers, MA, USA), anti-phospho (Tyr397)-FAK (#8556, Cell Signaling Technology, Danvers, MA, USA), anti-FAK (#13009, Cell Signaling Technology, Danvers, MA, USA), anti-phospho-4E-BP1 (Thr37/46) (# 9459, Cell Signaling Technology, Danvers, MA, USA), anti-4E-BP1 (# 9452, Cell Signaling Technology, Danvers, MA, USA), HA.11 clone 16B12 (1:1000; BioLegend, California, USA), anti-β-actin (#47778, Santa Cruz Biotechnology, CA, USA), and anti-GAPDH (ab8245, Abcam, Ontario, Canada).

### Cell culture, transfection, and Lentivirus production and infection

Clonetics™ Prostate Epithelial cells (PrEC) were cultured using the Prostate Epithelial Cell Medium BulletKit™ according to manufacturer instructions (Lonza, NJ, USA). Prostate cancer cells LNCaP (CRL-1740) and PC3 (CRL-1435) were cultured according to manufacturer instructions (American Type Culture Collection, VA, USA). DU145 (HTB-81), were cultured in RPMI 1640 (Wisent Bioproducts, Quebec, Canada) supplemented with 10% HyClone FBS and 1% HyClone Penicillin-Streptomycin (Thermo Fisher Scientific, Ontario, Canada). HEK 293 T cells (CRL-3216) were cultured according to manufacturer instructions (American Type Culture Collection, VA, USA). All media was supplemented with MycoZap™ Prophylactic (Lonza, NJ, USA) and were routinely checked for mycoplasma. All cell cultures were incubated at 37 °C, 5% CO_2_.

Lentiviral particles were produced in HEK-293 T cells and harvested as previously described [37]. Cells were infected twice with equal volumes of virus and media containing 8 μg/ml Hexadimethrine Bromide (Sigma-Aldrich, Ontario, Canada). Cells were collected 48 h post-secondary infection.

### Western blotting

Cells were lysed in RIPA buffer as previously described [45], sonicated and centrifuged (13,000 rpm, 10 min or 1200 g, 10 min for phospho-proteins). Protein concentration was determined with the Bicinchoninic Acid protein assay kit (Thermo Fisher Scientific, Ontario, Canada) and 10–50 μg of protein was separated by SDS-PAGE and transferred onto nitrocellulose membranes (Bio-Rad, Ontario, Canada). Membranes were then blocked and incubated with antibodies as previously described [45].

### RNA extraction, reverse transcription, and real-time quantitative PCR

RNA extraction, reverse transcription and qRT-PCR were performed as previously described [45]. Primer sequences were as follows: Staufen1 (fwd 5′-AACGGAACTTGCCTGTGAAT-3′, rev 5′-AGGGGCGGTAACTTCTTCAG-3′) and, GAPDH (fwd 5′-AACCACAGTCCATGCCATCAC-3′, rev 5′-TCCACCACCCTGTTGCTGTA-3′).

### Immunofluorescence

For immunofluorescent staining, cells were fixed for with 4% formaldehyde in 1X PBS pH 7.4 for 30 min and then permeablized with 0.5% Triton X-100 in 1X PBS pH 7.4 for 15 min. Cells were then blocked with 10% fetal bovine serum (FBS) for 1 h at room temperature. Cells were incubated with the primary antibody diluted in 1% FBS, 0.1% Triton X-100 overnight at 4 °C. Next the cells were washed 3 × 5 min with 1X PBS pH 7.4 and then incubated with Alexa secondary antibodies (Invitrogen, Ontario, Canada) for 1–2 h at room temperature. Cells were washed 3 × 5 min with 1X PBS pH 7.4 and then mounted on slides using Vectashield mounting media containing DAPI for staining nuclei (Vector Labs, Ontario, Canada). Cells were visualized by microscopy using a Zeiss AxioImager.M2 microscope.

### Proliferation assay and cell cycle analysis

Cell proliferation was quantified by flow cytometry using 5-bromo-2′-deoxyuridine (BrdU) incorporation. Cells were seeded on 60 mm culture plates (3 × 10^5^ for DU145 and LNCaP and 4 × 10^5^ for PC3), infected with CTL or Staufen1-shRNA lentivirus and maintained at 70% confluency. BrdU (30 μM) (Invitrogen, Ontario, Canada) was added to cultures 48 h post-secondary infection and incubated for 2 h. Cells were stained as previously described [37]. Stained cells were analyzed using the BD FACSCelesta™ or the BD LSRFortessa™ flow cytometers. Data analysis was performed using FlowJo software.

### Apoptosis assay

Cells were seeded in 60 mm culture plates (as stated above), infected with CTL or Staufen1-shRNA lentivirus and maintained at 70% confluency. Cells were co-stained 48 h post-secondary infection using the Alexa Fluor_®_ 488 Annexin V/Dead Cell Apoptosis Kit with Alexa Fluor_®_ 488 annexin V and PI for Flow Cytometry (Thermo Fisher Scientific, Ontario, Canada) according to manufacturer instructions. Stained cells were analyzed using the BD FACSCelesta™ or the BD LSRFortessa™ flow cytometers. Data analysis was performed using FlowJo software.

### Motility and invasion assay

Infected cells (2.5 × 10^4^) were seeded in serum free medium in Corning_®_ BioCoat™ Control Inserts (#354578) or Corning_®_ GFR Matrigel_®_ Basement Membrane Matrix Invasion Chambers (#354480) (VWR International, Ontario, Canada) containing growth medium in the bottom chamber. DU145 and PC3 cells were incubated for 24 and 72 h, respectively. Cells were fixed and stained with the Kwik-Diff™ Stain (Thermo Fisher Scientific, Ontario, Canada). Cell motility and invasion were assessed according to manufacturer instructions. Five random fields of view were imaged and analyzed using Northern Eclipse Software (NES, Expix Imaging, Ontario, Canada).

### Migration assay

DU145 and PC3 cells were infected with CTL or Staufen1-shRNA lentivirus for 48 h post-secondary infection and 70ul was seeded into culture inserts (DU145 CTL 8 × 10^5^ cells/ml; DU145 shStau1 1 × 10^6^ cells/ml; PC3 CTL 4 × 10^5^; shStau1 4 × 10^5^ cells/ml) to form a confluent monolayer (Ibidi, Munich, Germany). Inserts were removed 24 h post-seeding and washed with PBS and imaged in real time for up to 24 h using the Incucyte_®_ ZOOM system (Essen Bioscience, MI, USA). The gap was measured using Northern Eclipse Software (NES, Expix Imaging, Ontario, Canada). The average distance at 3 points was calculated. These values were normalized to the % confluence of cells.

### Statistical analysis

All experiments were performed with a minimum of *n* ≥ 3 biological replicates unless otherwise stated. The data were analyzed using the student’s t-test or a one-sample t-test as indicated. Significance was set at *P* ≤ 0.05 with *P ≤ 0.05, **P ≤ 0.01, and ***P ≤ 0.001. Error bars represent standard deviation (SD).

## Results

### Staufen1 is increased in human prostate Cancer

We examined Staufen1 levels across three separate PC cell lines, namely, PC3, DU145 and LNCaP cells, and compared its expression levels to those observed in normal prostate epithelial cells (PrEC) [59]. These cell lines are commonly used to decipher the mechanisms underlying PC since they represent both androgen-sensitive and -insensitive entities [60–62]. Specifically, PC3 cells were derived from a vertebral bone metastasis site and are classified as prostatic small cell neuroendocrine carcinoma and are androgen-insensitive [60–63]. DU145 cells are also androgen-insensitive and were isolated from brain metastasis of human prostate adenocarcinoma [60–62], while LNCaP cells were established from an androgen-sensitive metastatic lesion of human prostatic adenocarcinoma in the lymph nodes [60–62, 64].

**Fig. 1:**
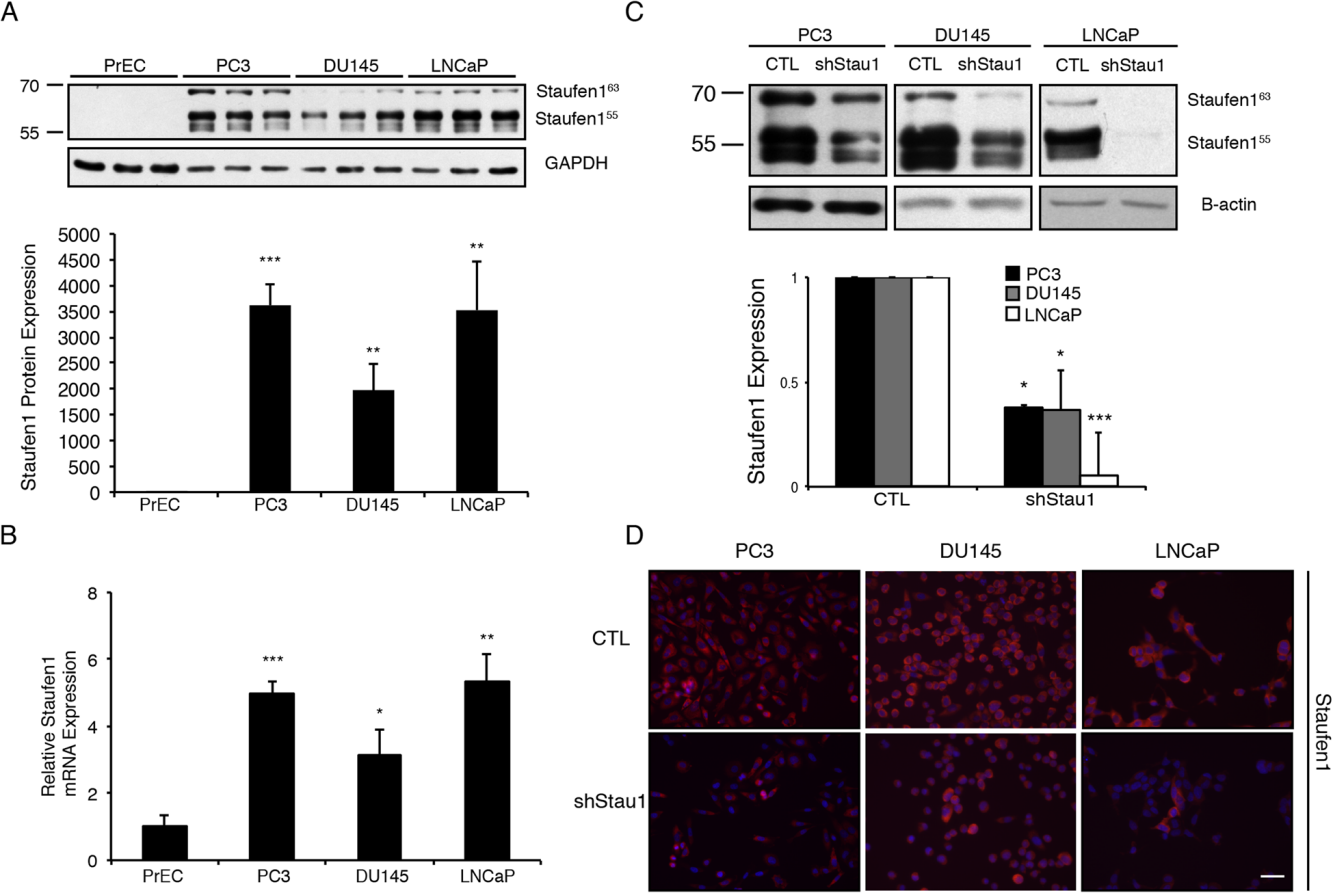
Staufen1 is overexpressed in prostate cancer cell lines. Expression of Staufen1 was determined in human prostate epithelial cells (PrEC), androgen-insensitive (PC3, DU145) and androgen-sensitive (LNCaP) prostate cancer cells. **a** Western blot using anti-Staufen1 antibodies and GAPDH as a loading control. Both Stau1 isoforms (55 and 63 kDa) were quantified (*n* = 3). **b** qRT-PCR analysis using primers specific for Staufen1 mRNA and normalized to total levels of GAPDH (n = 3). Quantifications are normalized relative to PrEC. **c** Representative western blots using anti-Staufen1 antibodies and B-actin as a loading control (n = 3) in PC3, DU145 and LNCaP cells. Each representative blot is cropped to show an *n* = 1 for each cell line from their respective full-length blots. Note that the LNCaP cells used for Fig. 1 experiments were also used for Figs. 2 and 3 and therefore a different representative image from the same blot was selected for Fig. 1. Quantification is normalized to CTL (n = 3). **d** Immunofluorescence of PC3, DU145 and LNCaP cells after 48 h of infection with Control (CTL) or Staufen1-shRNA (shStau1) lentivirus. Cells were stained with an anti-Staufen1 antibody (red) and nuclei with DAPI (blue), scale bars = 50 μm. Data are mean ± SD, Student’s T-Test in (**a**) and (**b**), One-sample T-Test in (**c**) *P < 0.05, **P < 0.01, ***P < 0.001. Full length blots are presented in Supplemental Fig. 3

Western blot and RT-qPCR were first performed to analyze Staufen1 protein and mRNA expression, respectively. Our results show that Staufen1 is markedly increased (*P* < 0.05) in all cell lines compared to PrEC at both protein and mRNA levels (Fig. 1a, b). In this context, the Human Protein Atlas [65] (v18.1.proteinatlas.org) also identifies Staufen1 as a prognostic factor in human prostate cancer where survival rates decrease in patients with high Staufen1 expression (*N* = 99) compared to those with low Staufen1 expression (*N* = 395) (Protein Atlas; Stau1).^1^ These findings are in agreement with the cell line data presented above and indicate that Staufen1 is also increased in human PC tumours.

### Staufen1 differentially regulates cell growth in prostate Cancer cell lines

In separate experiments, we assessed the efficiency of our Staufen1 shRNA lentivirus in all three PC cells lines (PC3, DU145, and LNCaP). Control (CTL) or Staufen1-targeting shRNA lentiviruses, using a mix of three independent Staufen1 targeting shRNAs, were used to transduce all three PC cell lines. To confirm Staufen1 knockdown in each cell line, we performed Western blot and immunofluorescence staining using anti-Staufen1 antibodies. Results showed high knockdown efficiency of ~ 60% for PC3 and DU145 cells (*P* < 0.05) and ~ 90% for LNCaP cells (*P* < 0.001) (Fig. 1c, d). In our hands, LNCaP cells displayed greater Staufen1 knockdown compared to DU145 and PC3 cells in this transient cell system. This discrepancy may be due to cell type specific properties as described above, which likely contribute to the varying level of transduction efficiencies.

**Fig. 2:**
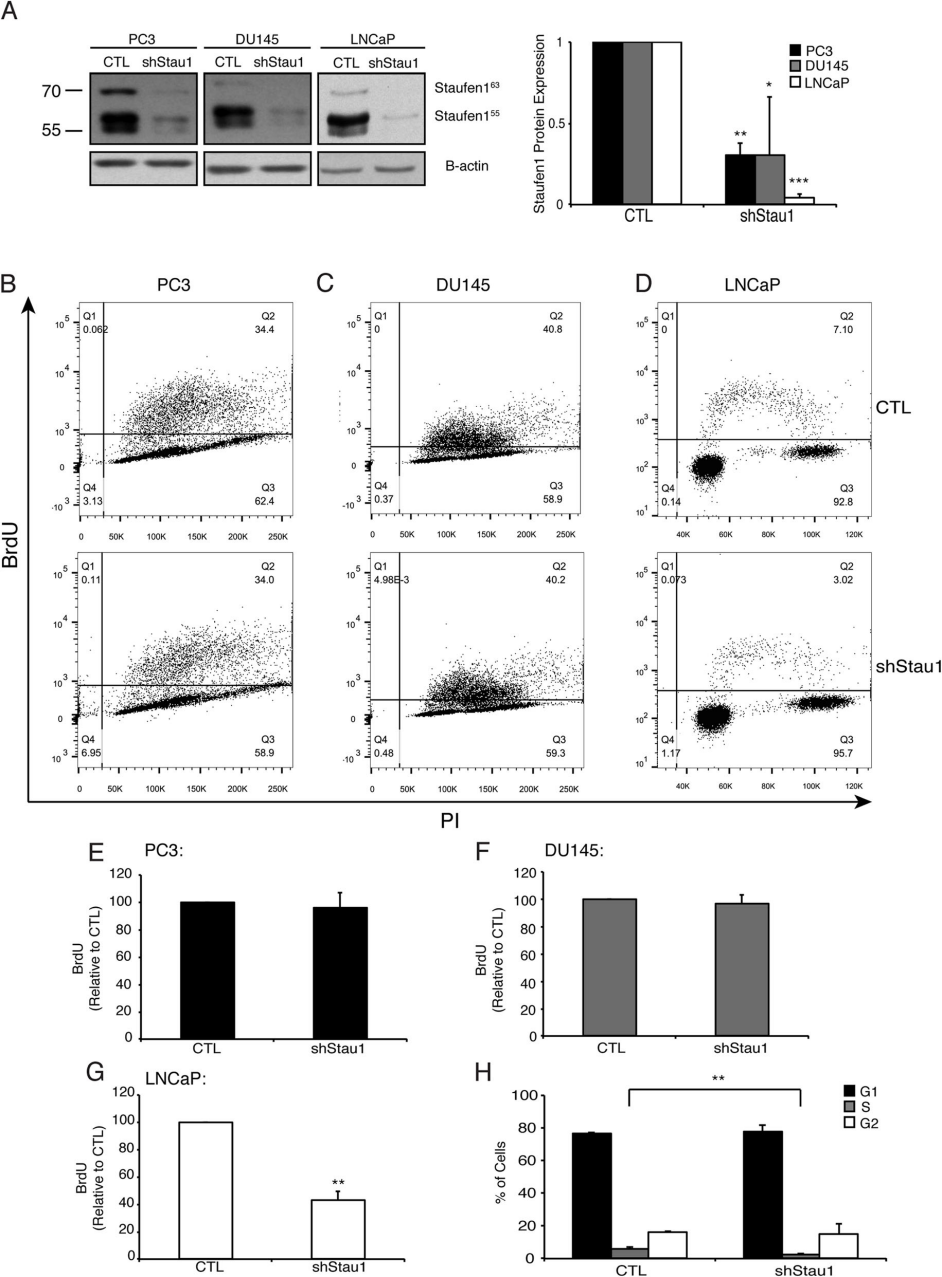
Staufen1 regulates the proliferation and cell cycle of LNCaP cells. Analysis of cell proliferation was performed after 48 h of Control (CTL) or Staufen1-shRNA expression (shStau1) in all three PC cell lines. **a** Representative western blot of Staufen1 expression with B-actin as a loading control in PC3, DU145, and LNCaP cells. Each representative blot is cropped to show an n = 1 for each cell line from their respective full-length blot. Note that the LNCaP cells used for Fig. 1 experiments were also used for Figs. 1 and 3 and therefore a different representative image from the same blot was selected for Fig. 2. Quantification is represented normalized to CTL (n = 3). **b-d** Proliferation was assessed by two-parameter flow cytometry and representative dot plots following 2 h of BrdU incorporation and Propidium Iodide (PI) staining in PC3, DU145, and LNCaP cells expressing CTL or Staufen1-shRNAs. **e-g** Quantification of BrdU incorporation is represented as a percentage relative to CTL in all three PC cell lines (n = 3). **h** the percentage of the cells in G1, S and G2 phases are indicated. Data are Mean ± SD, One-Sample T-Test, *P < 0.05, ** P < 0.01, ***P < 0.001. Full length blots are presented in Supplemental Fig. 4

With the establishment of this cell system, we next examined the role of Staufen1 on proliferation across the various PC cell lines. We evaluated cell proliferation using two-parameter flow cytometry to measure bromodeoxyuridine (BrdU) and propidium iodide (PI) staining. In these experiments, Western blot analysis confirmed Staufen1 knockdown in all cell lines, ~ 70% for PC3 and DU145 cells (*P* < 0.01, *P* < 0.05, respectively) and ~ 95% (*P* < 0.001) for LNCaP cells 48 h post-infection (Fig. 2a). Following Staufen1 knockdown, LNCaP cells showed an ~ 50% decrease in BrdU incorporation, which revealed their lower proliferative capacity (P < 0.01) as compared to CTL (Fig. 2d, g, h). By contrast, cell proliferation was unaffected (*P* > 0.05) in PC3 and DU145 cell lines (Fig. 2b, c, e, f).

**Fig. 3:**
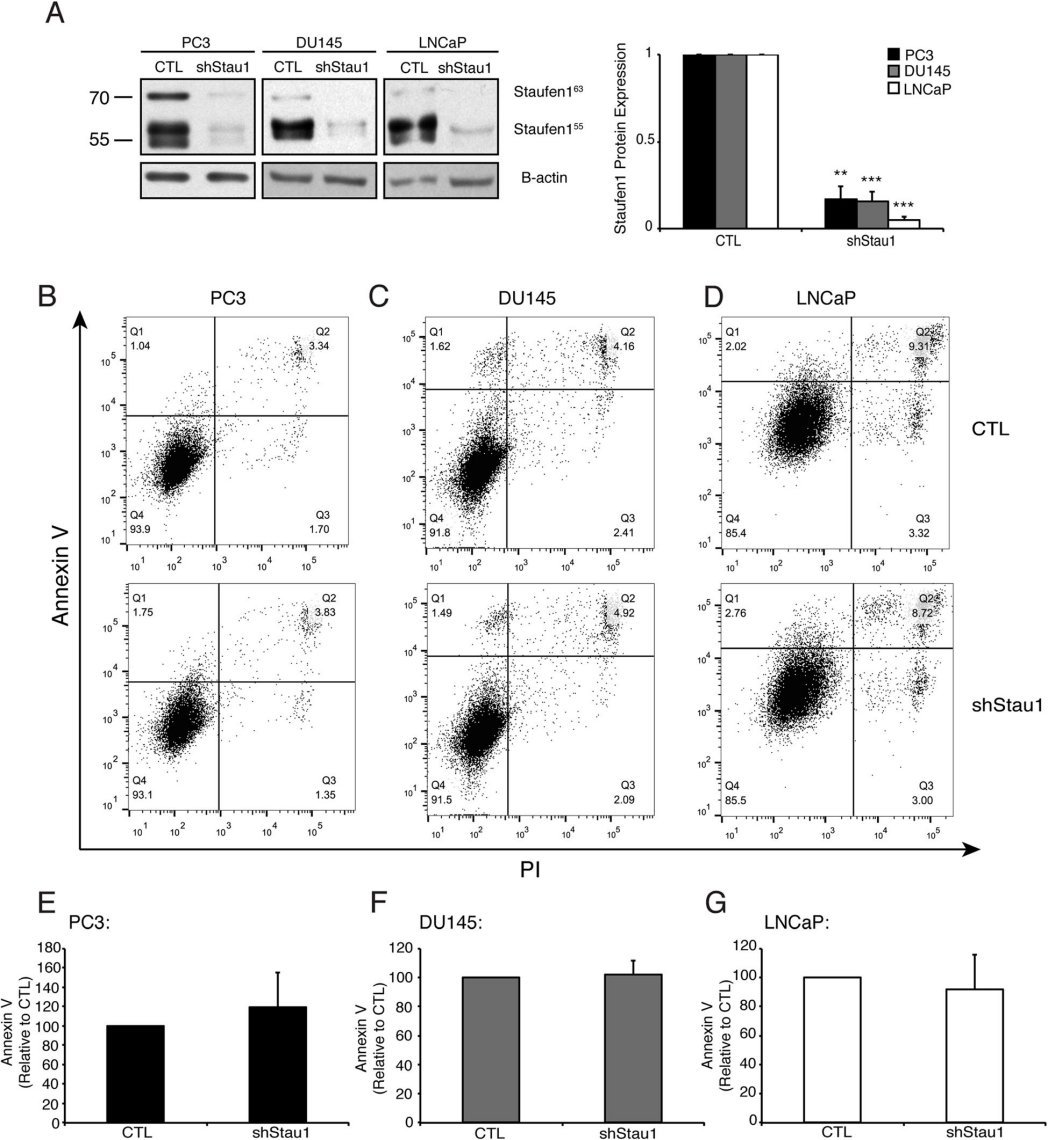
Staufen1 does not regulate apoptosis in Prostate Cancer cells. Analysis of apoptosis was performed after 48 h of Control (CTL) or Staufen1-shRNA expression (shStau1) in PC cells using Annexin V and Propidium Iodide (PI) dual staining. **a** Representative western blot of Staufen1 expression with B-actin as a loading control in PC3, DU145, and LNCaP cells. Each representative blot is cropped to show an n = 1 for each cell line from their respective full-length blot. Note that the LNCaP cells used for Fig. 3 experiments were also used for Figs. 1 and 2 and therefore a different representative image from the same blot was selected for Fig. 3. Quantification is represented normalized to CTL (n = 3). **b-d** Apoptosis was assessed by two-parameter flow cytometry and representative dot plots of CTL and Staufen1-shRNA expressing PC3, DU145, and LNCaP cells. **e-g** Quantification of Annexin V staining is represented as a percentage relative to CTL (n = 3). Data are Mean ± SD, One-Sample T-Test. Full length blots are presented in Supplemental Fig. 5

Subsequently, dual staining of cells with Annexin V and PI was performed and analyzed by flow cytometry to assess apoptosis in PC cells expressing Staufen1-shRNA. Western blot analysis demonstrated reduced Staufen1 levels in all cell lines with highly efficient knockdown (*P* < 0.01; Fig. 3a). Our data collectively show that apoptosis in all PC cell lines was unaffected (Fig. 3b-g). Overall, these data indicate that Staufen1 has differential roles in PC where it regulates proliferation in LNCaP but not in PC3 or DU145 cells, without impacting apoptosis in either form of PC.

### Staufen1 regulates cell migration and invasion in PC3 and DU145 prostate Cancer cells

To investigate the possible role of Staufen1 on invasion and migration properties of PC cells, we carried out transwell invasion and migration assays. We performed these experiments using PC3 and DU145 cell lines since they are derived from prostate cancer metastatic sites [60, 61, 66] and are highly invasive [15, 66]. LNCaP cells are non-metastatic [14, 15, 67], adhere loosely to the substrate, and have the capacity for anchorage-independent proliferation [61]. When cultured at high densities, which is required for these assays, LNCaP cells detach as sheets [61], making this cell line unreliable for migration and invasion assays.

**Fig. 4:**
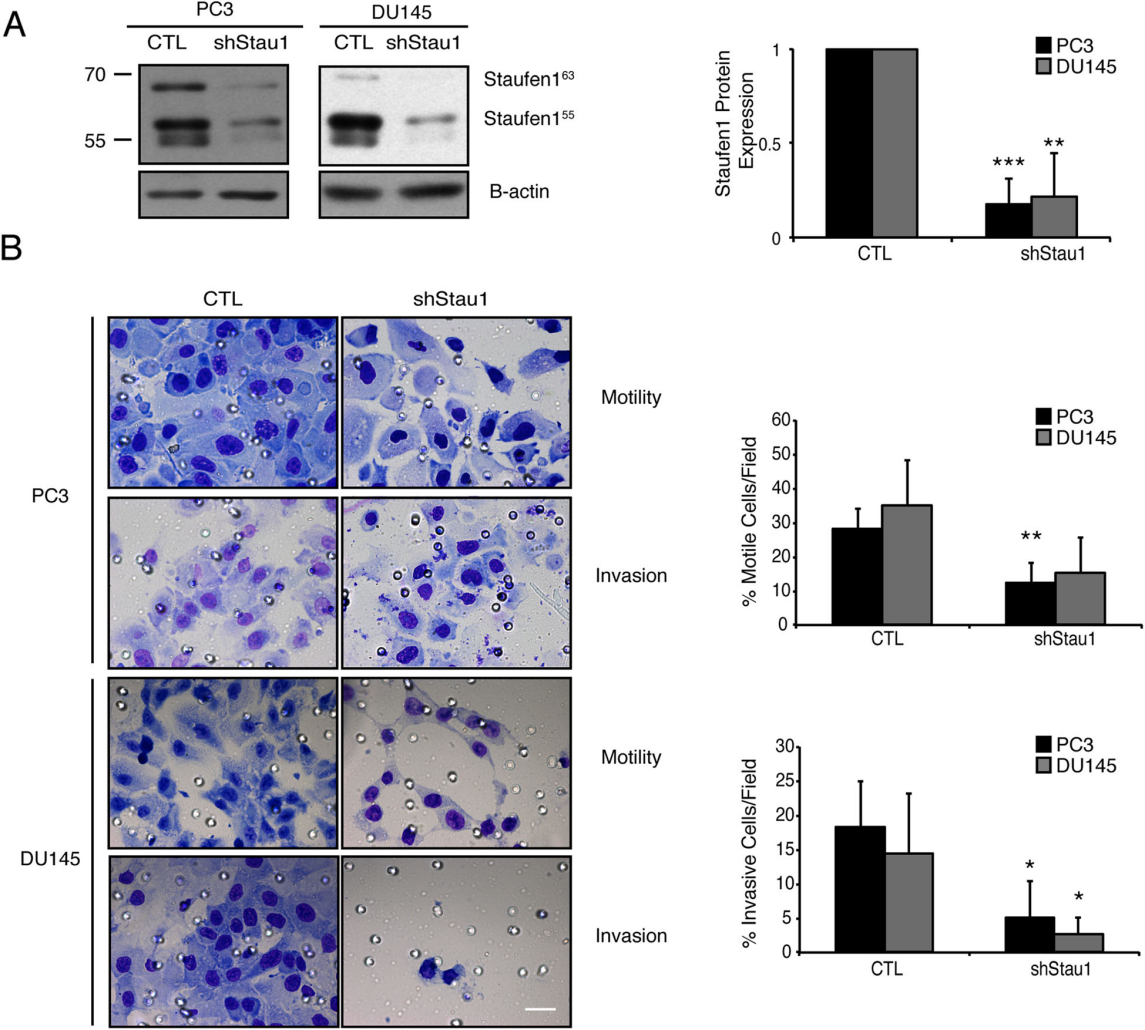
Staufen1 knockdown inhibits Prostate Cancer cell invasion and motility. Motility and Invasion assays were performed following 48 h of Control (CTL) or Staufen1-shRNA (shStau1) expression. **a** Western blot analysis of Staufen1 expression showing a representative blot of Staufen1 with β-actin as a loading control in PC3 and DU145 cells. Each representative blot is cropped to show an n = 1 for each cell line from their respective full-length blot. Note that the DU145 cells used for Fig. 4 were also used for Fig. 5 experiments and therefore a different representative image from the same blot was selected for each figure. Quantification of *n* = 4 is represented normalized to CTL. Data are Mean ± SD, One-Sample T-Test, **P < 0.01, ***P < 0.001. **b** Cells were seeded into transwell chambers containing membranes coated with or without Matrigel and incubated for 72 h and 24 h for PC3 and DU145 cells, respectively. Representative images of cells that passed through the transwell chamber at 40X magnification, scale bar = 20 μm, are displayed. Average number of cells/field of view is quantified for cell motility (no matrigel) and invasion (matrigel) as a percentage relative to CTL. Quantifications are for all data are *n* = 4. Data are Mean ± SD, Students T-Test, **P < 0.01, ***P < 0.001. Full length blots are presented in Supplemental Fig. 6

Western blotting confirmed Staufen1 knockdown (~ 80%, *P* < 0.01) in PC3 and DU145 cells (Fig. 4a). Cells were plated into transwell chambers with either a CTL porous membrane or matrigel-coated membrane. The knockdown of Staufen1 decreased cell motility by ~ 40% (P < 0.01 and *P* = 0.06) in PC3 and DU145 cells, respectively (Fig. 4b). Next, we assessed invasion in the presence of matrigel and observed that both PC3 and DU145 cell invasion levels were decreased by ~ 60% and ~ 80%, respectively upon Staufen1 knockdown (*P* < 0.05; Fig. 4b).

**Fig. 5:**
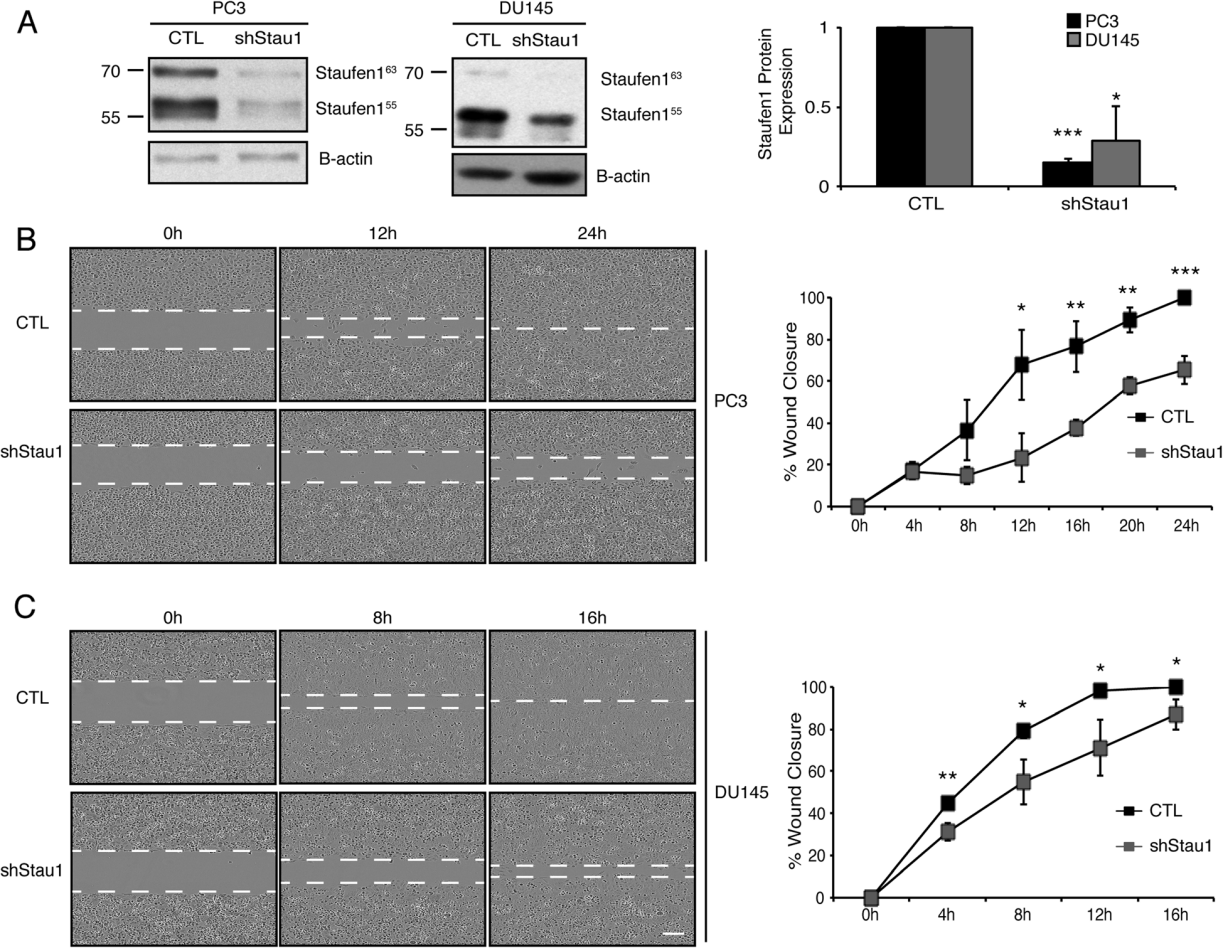
Staufen1 knockdown inhibits Prostate Cancer cell migration. Migration assays were performed after 48 h of Staufen1 knockdown and **a** is a representative western blot of Staufen1 expression with β-actin as a loading control in PC3 and DU145 cells. Each representative blot is cropped to show an n = 1 for each cell line from their respective full-length blot. Note that the DU145 cells used for Fig. 4 were also used for Fig. 5 experiments and therefore a different representative image from the same blot was selected for each figure. Quantification is represented normalized to CTL (n = 3). Data are Mean ± SD, One-Sample T-Test, **P < 0.01, ***P < 0.001. **b-c** Cells were seeded into culture insert wells for 24 h, following removal of insert, a 500 μm cell free gap represents 0 h, and consecutive photos were taken at 4 h intervals until gap closure was achieved in CTL cells. Representative images at 10X magnification, scale bar = 300 μm. Quantification of the gap is represented as % wound closure normalized to cell confluency. Quantifications are for all data are *n* = 3. Data are Mean ± SD, Students T-Test, *P < 0.05, **P < 0.01, ***P < 0.001. Full length blots are presented in Supplemental Fig. 7

Next, we performed migration assays to further evaluate the impact of Staufen1 on PC cell motility and migration. Western blotting confirmed Staufen1 knockdown of greater than 70% in PC3 cells and DU145 cells (*P* < 0.001 and P < 0.05, respectively; Fig. 5a). Cells were seeded into culture inserts and cultured for 24 h. A 500 μm cell free gap was created by removing the insert which represented time 0 h. Migration of the cells was subsequently evaluated at 4 h-intervals until 100% gap closure was achieved in CTL cells. Results of these experiments revealed that upon Staufen1 knockdown, PC3 and DU145 cells showed significant (P < 0.05) reductions in migration at almost all time points examined as compared to CTL cells (Fig. 5b, c). The fact that both cell motility and invasion were decreased in PC3 and DU145 cells with Staufen1 knockdown supports the notion that the overexpression of Staufen1 we observed in PC cancer cells (see Fig. 1) translates into marked increases in metastasis capacity.

### Staufen1 differentially regulates tumorigenesis via regulation of signaling pathways across prostate Cancer cell lines

**Fig. 6:**
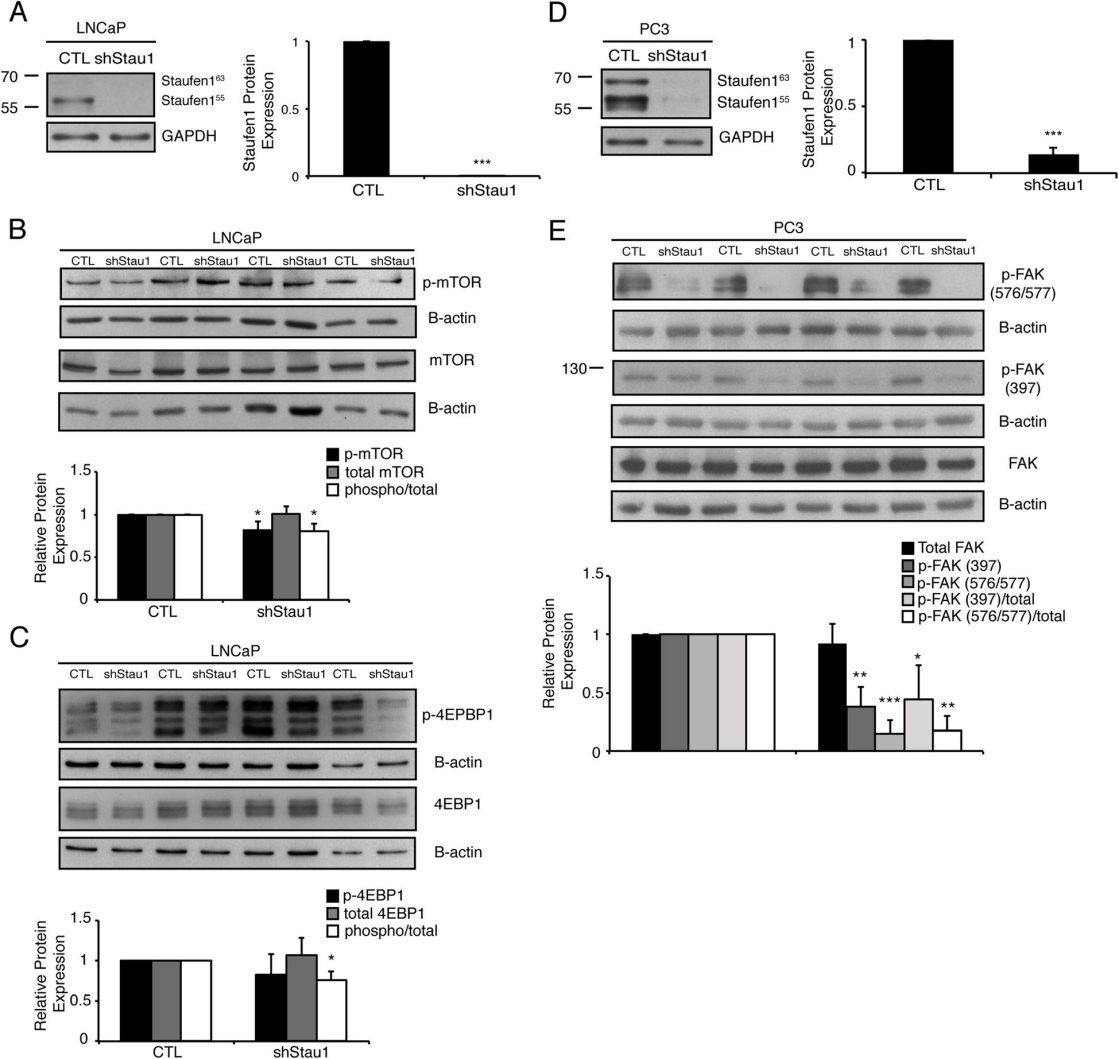
Staufen1 Differentially Regulates Tumorigenesis via regulation of Signaling Pathways across Prostate Cancer Cell Lines. Western blot analysis was performed 48 h post-infection of prostate cancer cells with Control (CTL) or Staufen1-shRNA (shStau1) lentivirus (**a** and **d**) representative Staufen1 expression in LNCaP and PC3 cells, respectively. LNCaP cells were examined for (**b**) expression of total mTOR and phospho-mTOR (Ser2448) and (**c**) expression of total 4E-BP1 and phospho-4E-BP1 (Thr37/46). PC3 cells were analyzed for members of the FAK signaling pathway where (**e**) represents total FAK and phospho-FAK (Tyr397 and Tyr576/577). All quantifications are normalized to loading controls GAPDH or β-actin and represented as a fold change relative to the Control (CTL) with n = 4. Data are Mean ± SD, One-Sample T-Test, *P < 0.05, **P < 0.01, ***P < 0.001. Note that each representative blot for Staufen1 in LNCaP, DU145 and PC3 cells is cropped to show an n = 1 for the respective cell lines from their full-length blots. Full length blots are presented in Supplemental Fig. 8 and Fig. 9

A key regulator of cell proliferation and growth is the mammalian target of rapamycin (mTOR) [28–30, 32, 34]. The mTOR kinase is central to two distinct complexes, mTORC1 and mTORC2 [68–71]. Upon activation, mTORC1 regulates cell growth and proliferation by modulating protein synthesis via phosphorylation of 4E-BP1 and S6K1 [68–72]. In order to determine whether mTOR is responsible for the reduced cell proliferation observed in LNCaP cells expressing shStau1, we examined the levels of activated mTOR. Western blot analysis confirmed Staufen1 knockdown (~ 95%; *P* < 0.001) in LNCaP cells 48 h post-infection (Fig. 6a). LNCaP cells expressing shStau1 showed a decrease (*P* < 0.05) of ~ 20% in phosphorylated mTOR relative to total mTOR as compared to CTL (Fig. 6b). Furthermore, 4E-BP1, a downstream target of mTOR, showed an ~ 25% decrease (P < 0.05) in phosphorylation relative to total 4E-BP1, compared to CTL cells (Fig. 6c). In contrast to these data, and in agreement with the lack of a change in proliferation, PC3 and DU145 cell lines, showed no alteration in mTOR activation in response to Staufen1 knockdown (*P* > 0.05; Supplemental Fig. 1). These data indicate that Staufen1 alters cell proliferation specifically in the LNCaP cell line via regulation of mTOR signaling through unknown mechanisms at this time.

The most prevalent and main complication in CRPC progression is bone lesion metastasis [3, 73–76]. Approximately two-thirds of all bone metastases are located in the spine [76]. Hence, we focused our efforts on determining the potential mechanisms by which Staufen1 regulates cell invasion in PC3 cells, which are derived from a bone metastatic site [60–62]. FAK is involved in the key aspects of the metastatic process and FAK signaling is crucial for focal adhesion turnover by regulating disassembly of focal adhesions, thereby highlighting a key role in cell migration and invasion [19, 77–80]. Following integrin clustering, FAK is recruited to the cytoplasmic portion of the β integrin subunits and is subsequently autophosphorylated at tyrosine 397, which causes kinase activation and creates a SH2 binding site for the protein tyrosine kinase c-Src [81, 82]. Upon adhesion, Src phosphorylates tyrosines 567 and 577 within the kinase domain of FAK to stimulate maximal FAK activity [81, 82].

As mentioned above, shStau1 expressing PC3 cells showed reduced invasion and migration relative to CTL conditions. Therefore, we examined key members of the FAK signaling family to investigate potential mechanisms by which Staufen1 impacts cell migration and invasion. Western blot analysis confirmed Staufen1 knockdown (~ 85%; *P* < 0.001) in PC3 cells, 48 h post-infection (Fig. 6d). Cells with reduced Staufen1 expression showed a decrease of ~ 55% (*P* < 0.05) in the ratio of phosphorylated Tyr397 FAK/total FAK and ~ 85% (*P* < 0.01) in phosphorylated Tyr576/577 relative to total FAK, as compared to CTL conditions (Fig. 6e). These data indicate that Staufen1 overexpression plays a role in the activation of FAK in the metastatic PC3 cell line, thus impacting cell motility.

Next, we attempted to overexpress Staufen1-HA in the knockdown cell lines to determine if ectopic Staufen1 expression could rescue the observed impact of the knockdown on migration and invasion in PC3 and DU145 cell lines. As shown in Supplemental Fig. 2, we were not able to achieve overexpression of Staufen1-HA in the knockdown PC cell lines as the Staufen1-shRNAs likely target both endogenous and overexpressed Staufen1-HA.

## Discussion

The high mortality rate associated with CRPC is a result of the aggressive nature of PC cells in forming metastatic lesions [6, 10, 12, 13]. Despite recent advances in our understanding of the underlying molecular mechanisms, prognosis remains poor for advanced PC [5], highlighting the need for additional investigation focused on the progression of CRPC. Our recent work has focused on Staufen1, a multi-functional double-stranded RBP with several roles in post-transcriptional regulation of gene expression [38–40, 42, 43, 45–51, 83, 84]. Multiple lines of evidence show that Staufen1 has important regulatory roles during skeletal muscle differentiation [41, 42, 45, 85–88] and that it is misregulated in various neuromuscular disorders [49, 51, 89, 90]. Its role in cancer biology has only recently begun to emerge, yet its implication in specific cancers including prostate cancer, deserves attention.

Cell motility, invasion and proliferation are key hallmarks of metastatic cancer, and elucidating the molecular mechanisms underlying these tumorigenic properties is crucial for the development of novel therapeutic strategies for these devastating diseases. In the current study, we investigated how Staufen1 regulates motility, invasion and proliferation in commonly studied prostate cancer cells. Our data show that Staufen1 is highly increased in PC and that this misregulation differentially impacts the various cell lines by regulating either cell growth or migration/invasion. Specifically, we report that Staufen1 regulates the proliferation of LNCaP cells via the mTOR signaling pathway. Interestingly, mTOR is a protein kinase that controls several anabolic processes required for cell growth and proliferation [71, 72]. Misregulation of mTOR signaling has been previously implicated in various forms of cancer [35, 71, 72, 91, 92]. In PC, 70–100% of cases of advanced disease are characterized by misregulation of the PI3K/AKT/mTOR signaling pathways [31]. Such misregulation has been associated with resistance to androgen deprivation therapy, disease progression, and poor outcomes [31]. A key aspect of CRPC progression is persistent androgen receptor (AR) signaling [93]. One mechanism by which PC bypasses the need for androgen is through gain-of-function mutations in the androgen signaling pathway [10, 74]. Recent studies have demonstrated a direct link between PI3K/AKT/mTOR and AR signaling, revealing important cross-talk between these pathways during the development of androgen insensitivity [93]. Altered PI3K/AKT/mTOR signaling affects AR signaling and thus, reduces the intrinsic need for androgens to drive PC growth, further contributing to castration resistance [93]. Since androgen-insensitive cells, DU145 and PC3, have mutations in the AR, they likely have over-active mTOR signaling, unable to be regulated by Staufen1 [59, 60, 94].

mTOR is the catalytic subunit for two functionally distinct protein complexes known as mTORC1 and mTORC2 [71, 93]. Since mTORC1 is hyperactivated in a large subset of cancers, the regulation of mTOR activity is a promising therapeutic target and, accordingly, several small molecules have been developed to inhibit its activity in various forms of cancer [71], including rapalogs and second-generation mTOR inhibitors [95–106]. Altogether, these findings support the notion that regulation of mTOR signaling in PC is a viable therapeutic approach. Our data indicate that Staufen1 plays a key role in the activation of mTOR in LNCaP cells highlighting its potential as a novel therapeutic target to modulate mTOR activation in androgen-sensitive PC.

Conversely, in PC3 cells the knockdown of Staufen1 reduced motility and invasion but not proliferation through decreased activation of FAK. A complication of CRPC is the formation of metastatic lesions, primarily metastasizing to bone [3, 59, 60, 62, 73–76]. Notably, integrins and FAK signaling have been implicated in metastasis of PC to bone [107]. Cell migration and invasion are essential components of tumour metastasis and focal adhesions that drive metastatic characteristics in malignant cells [79, 107]. In addition, increased FAK activity and phosphorylation are associated with tumorigenesis in various forms of cancer [77–81]. Here, we demonstrate that Staufen1 regulates the phosphorylation of FAK in bone metastatic PC3 cells, therefore controlling cell invasion and migration properties in our cultured cell systems.

In several forms of cancer, including metastatic PC, FAK expression is upregulated and is associated with poor prognosis due to its role in metastasis and invasion [77, 78, 82, 108–110]. Several lines of evidence indicate that FAK-mediated signaling is involved in the development of tumour malignancy, therefore, several therapeutic approaches have been developed to modulate FAK activity [81]. In agreement with our data that show decreased migration/invasion in parallel to downregulated FAK activity in PC3 cells, several other studies have shown that a reduction in FAK expression and/or activity decreased the migration/invasion of cancer cells, including in PC [111–119]. Taken together with our results, these data further highlight the therapeutic potential of Staufen1 for PC and demonstrate its important function in metastasis of highly invasive PC cells through its regulation of FAK activity. The fact that Sugimoto et al. identified Staufen1-binding sites (SBS) in several mRNAs encoding proteins involved in FAK signaling reinforces the concept of a functional regulatory link between Staufen1 and FAK signaling [46]. More specifically, multiple integrin subunit mRNAs that are upstream of FAK, including A2, A3, A5, B1 and B8, all have SBS [46]. In addition, SHP-2, a protein-tyrosine phosphatase that can dephosphorylate FAK [120], and Src, a protein-tyrosine kinase which phosphorylates FAK [121, 122], also contain SBS [46]. These data are in fact well aligned with the known multi-functional nature of Staufen1 and suggest that Staufen1 regulates FAK signaling through several direct mRNA targets. Based on these observations, it will be important to determine the precise mechanism of action by which Staufen1 regulates invasion and migration of PC cells through these SBS-target mRNA interactions.

## Conclusions

Given our current and previous findings, as well as the few reports collectively showing the impact of Staufen1 in cell proliferation, apoptosis, migration and invasion, it seems that a clearer picture is beginning to emerge on the key role of Staufen1 in cancer biology and tumorigenesis. Furthermore, it appears that the misregulation of Staufen1 in cancer cells results in Staufen1 assuming different functions and triggering distinct signaling pathways according to specific disease contexts and cell types. This is perhaps not entirely surprising given the multi-functional nature of Staufen1 in different cells and at different stages of maturation. The complexity related to Staufen1-regulated cellular events, particularly in cancer, clearly warrants further studies to unravel its full potential and relevance as a valid biomarker and novel therapeutic target for a wide array of cancers.

## Supplementary Information

**Additional file 1: Supplemental Figure 1**. Western blot analysis was performed 48 hours post-infection of prostate cancer cells with Control (CTL) or Staufen1-shRNA (shStau1) lentivirus (A) Staufen1 in DU145 cells, (B) expression of total mTOR and phospho-mTOR (Ser2448) in DU145 cells, (C) expression of total mTOR and phospho-mTOR (Ser2448) in PC3 cells. All quantifications are normalized to loading controls GAPDH or β-actin and represented as a fold change relative to the Control (CTL) with n=4. Data are Mean ± SD, *P<0.05, **P<0.01, ***P<0.001. Note that each representative blot for Staufen1 in DU145 cells is cropped to show an n=1 for the respective cell lines from their full-length blots. The level of Staufen1 knockdown for PC3 cells is in Fig. 6d.

**Additional file 2: Supplemental Figure 2**. Western blot analysis was performed on Control (CTL) and Staufen1-shRNA (shStau1) expressing (A) DU145 and (B) PC3 prostate cancer cell lines that were subsequently infected with lentivirus encoding an empty vector (pcDH) or Staufen1-HA overexpression vector (pcDH-Staufen1-HA) following 24 and 48-hours post-infection. Western blots for total Staufen1, ectopic Staufen1-HA (HA) with the respective β-actin as a loading control.

**Additional file 3: Supplemental Figure 3**. Full length uncropped Western blots for Fig. 1. (A) Western blot using anti-Staufen1 and (B) GAPDH antibodies on PrEC, PC3, DU145 and LNCaP cells (n=3). (C) Full length western blot using anti-Staufen1 and B-actin antibodies on PC3 cells (n=3). (D) Full length western blot using anti-Staufen1 and (E) B-actin antibodies on LNCaP cells (n=3). (F) Full length western blot using anti-Staufen1 and (G) B-actin antibodies on DU145 cells (n=3).

**Additional file 4: Supplemental Figure 4**. Full length uncropped Western blots for Fig. 2. (A) Full length western blot using anti-Staufen1 and (B) B-actin antibodies on DU145 and PC3 cells (n=3). (C) Full length western blot using anti-Staufen1 and (D) B-actin antibodies on LNCaP cells (n=3).

**Additional file 5: Supplemental Figure 5**. Full length uncropped Western blots for Fig. 3. (A) Full length western blot using anti-Staufen1 and (B) B-actin antibodies on PC3 cells (n=3). (C) Full length western blot using anti-Staufen1 and (D) B-actin antibodies on DU145 cells (n=3). (E) Full length western blot using anti-Staufen1 and (F) B-actin antibodies on LNCaP cells (n=4).

**Additional file 6: Supplemental Figure 6**. Full length uncropped Western blots for Fig. 4. (A) Full length western blot using anti-Staufen1 and (B) B-actin antibodies on PC3 cells (n=4). (C) Full length western blot using anti-Staufen1 and (D) B-actin antibodies on DU145 cells (n=4). Note: DU145 cell lines used are the same as those presented in Supplemental Figure 6, as the same batch of cells were split and used for both sets of experiments.

**Additional file 7: Supplemental Figure 7**. Full length uncropped Western blots for Fig. 5. (A) Full length western blot using anti-Staufen1 and (B) B-actin antibodies on PC3 cells (n=3). (C) Full length western blot using anti-Staufen1 and (D) B-actin antibodies on DU145 cells (n=3). Note: DU145 cell lines used are the same as those presented in Supplemental Figure 5, as the same batch of cells were split and used for both sets of experiments.

**Additional file 8: Supplemental Figure 8**. Full length uncropped Western blots for Fig. 6. (A) Full length western blot using anti-Staufen1 and (B) GAPDH antibodies on LNCaP cells (n=4). (C) Full length western blot using anti-mTOR and (D) B-actin antibodies on LNCaP cells (n=4). (E) Full length western blot using anti-phospho-mTOR and (F) B-actin antibodies on LNCaP cells (n=4). (G) Full length western blot using anti-4EBP1 and (H) B-actin antibodies on LNCaP cells (n=4). (I) Full length western blot using anti-phospho-4EBP1 and (J) B-actin antibodies on LNCaP cells (n=4).

**Additional file 9: Supplemental Figure 9**. Full length uncropped Western blots for Fig. 6. (A) Full length western blot using anti-Staufen1 and (B) GAPDH antibodies on PC3 cells (n=4). (C) Full length western blot using anti-phospho(576/577)-FAK and (D) B-actin antibodies on PC3 cells (n=4). (E) Full length western blot using anti-phospho(397)-FAK and (F) B-actin antibodies on PC3 cells (n=4). (G) Full length western blot using anti-FAK and (H) B-actin antibodies on PC3 cells (n=4).

## Data Availability

The datasets used and/or analysed during the current study are available from the corresponding author on reasonable request.

## Abbreviations

PC: Prostate cancer
PIN: Prostate intraspithelial neoplasia
CRPC: Castration-resistant prostate cancer
FAK: Focal adhesion kinase
RBP: RNA-binding protein
RMS: Rhabdomyosarcoma
PrEC: Prostate epithelial cells
FBS: Fetal bovine serum
BrdU: 5-bromo-2′-deoxyuridine
SEM: Standard error of the mean
RT-qPCR: Real time quantitative PCR
CTL: Control
PI: Propidium iodide
mTOR: Mammalian target of rapamycin
SBS: Staufen-1 binding site
AR: Andogren receptor
shStau1: Staufen-1 shRNA
Ser: Serine
Thr: Threonine
Tyr: Tyrosine

## Footnotes

1 https://www.proteinatlas.org/ENSG00000124214-STAU1/pathology
