# Supplementary figures and images for: Distinct roles for the RNA-binding protein Staufen1 in prostate cancer
Source: BMC Cancer. 2021 Feb 4;21:120. doi: 10.1186/s12885-021-07844-2 (PMC7863451; doi:10.1186/s12885-021-07844-2)

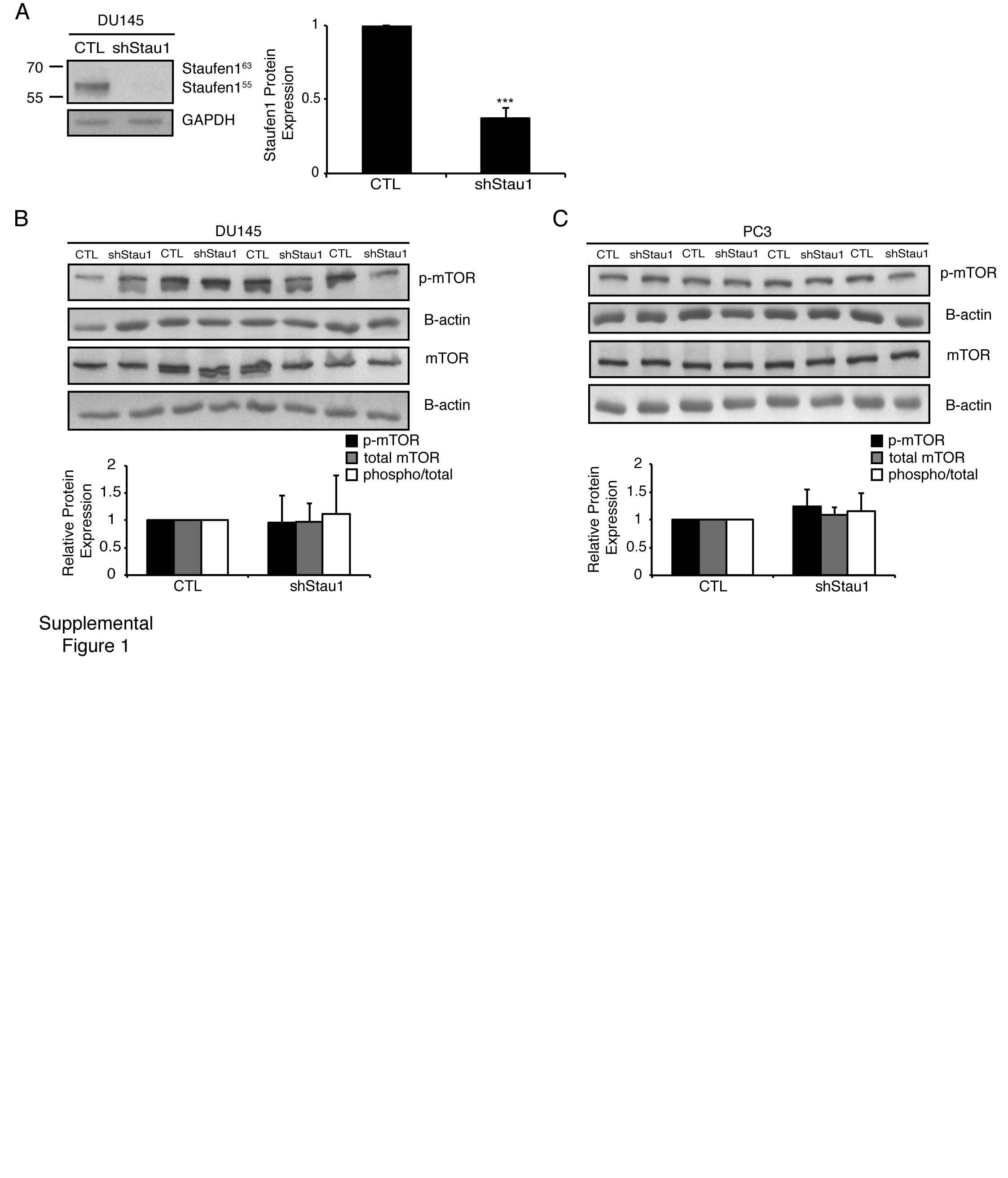

Supplement: Supplementary file 1 — Additional file 1: Supplemental Figure 1. Western blot analysis was performed 48 hours post-infection of prostate cancer cells with Control (CTL) or Staufen1-shRNA (shStau1) lentivirus (A) Staufen1 in DU145 cells, (B) expression of total mTOR and phospho-mTOR (Ser2448) in DU145 cells, (C) expression of total mTOR and phospho-mTOR (Ser2448) in PC3 cells. All quantifications are normalized to loading controls GAPDH or β-actin and represented as a fold change relative to the Control (CTL) with n=4. Data are Mean ± SD, *P<0.05, **P<0.01, ***P<0.001. Note that each representative blot for Staufen1 in DU145 cells is cropped to show an n=1 for the respective cell lines from their full-length blots. The level of Staufen1 knockdown for PC3 cells is in Fig. 6d. [file 12885_2021_7844_MOESM1_ESM.tif]

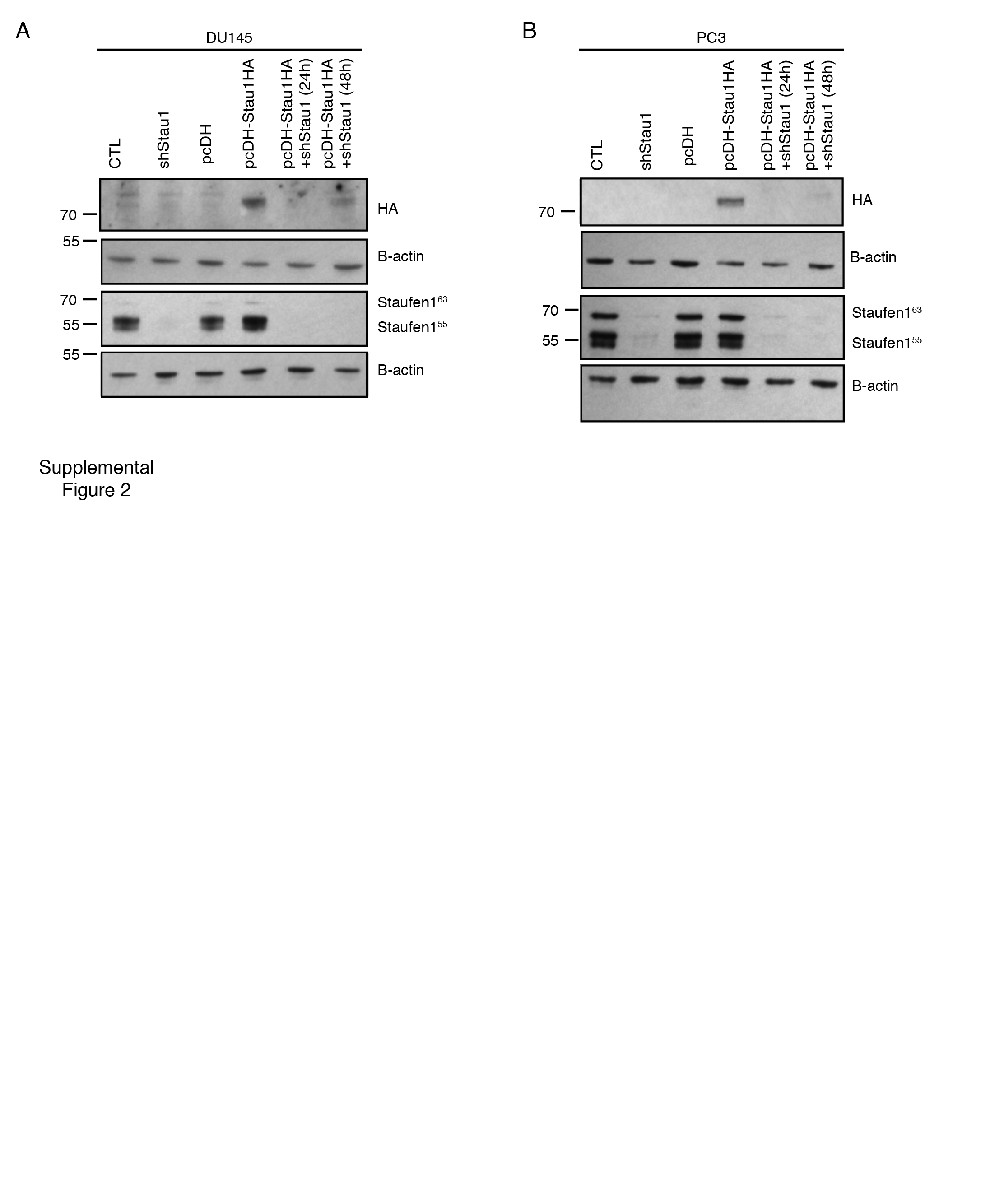

Supplement: Supplementary file 2 — Additional file 2: Supplemental Figure 2. Western blot analysis was performed on Control (CTL) and Staufen1-shRNA (shStau1) expressing (A) DU145 and (B) PC3 prostate cancer cell lines that were subsequently infected with lentivirus encoding an empty vector (pcDH) or Staufen1-HA overexpression vector (pcDH-Staufen1-HA) following 24 and 48-hours post-infection. Western blots for total Staufen1, ectopic Staufen1-HA (HA) with the respective β-actin as a loading control. [file 12885_2021_7844_MOESM2_ESM.tif]

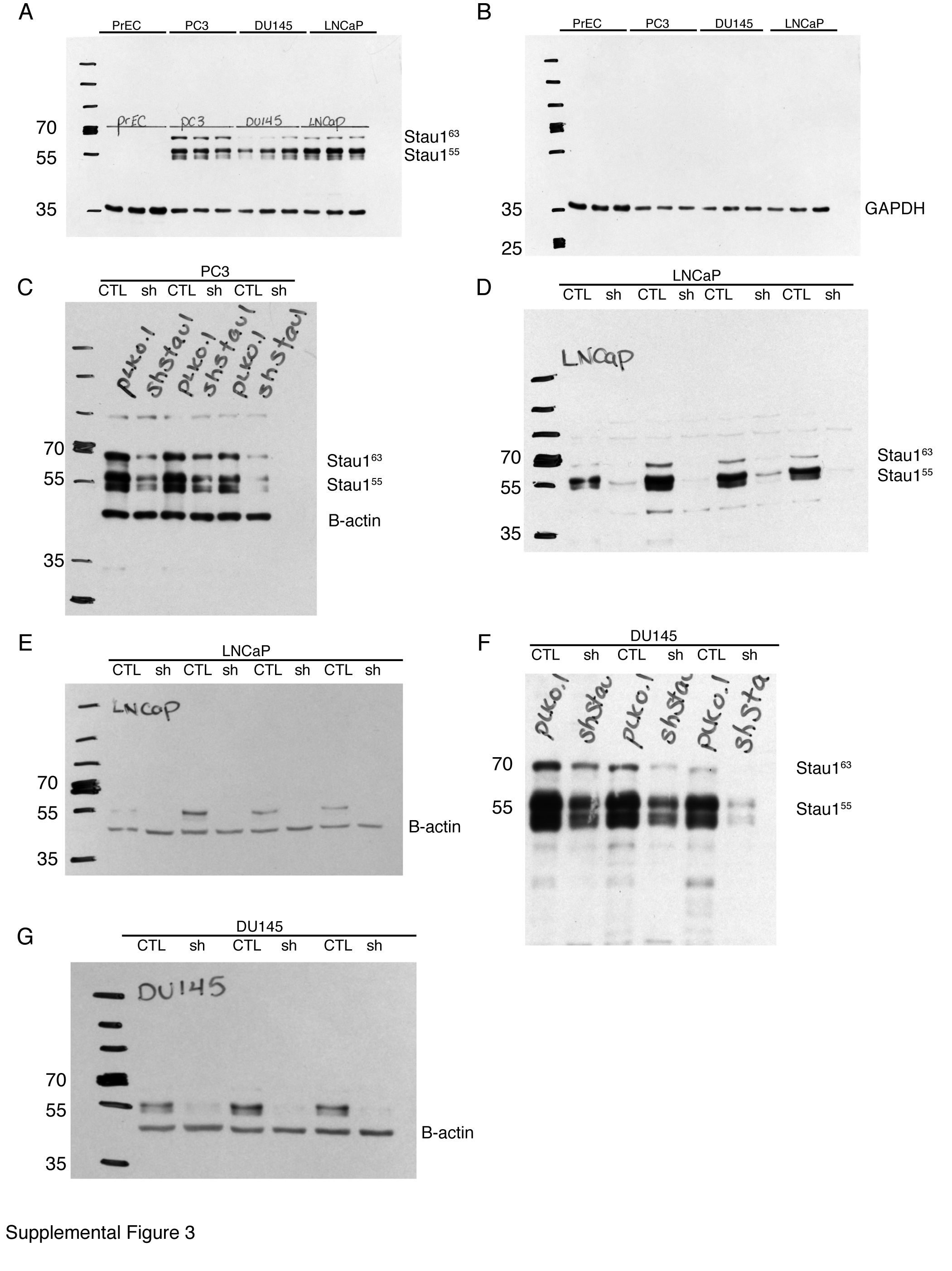

Supplement: Supplementary file 3 — Additional file 3: Supplemental Figure 3. Full length uncropped Western blots for Fig. 1. (A) Western blot using anti-Staufen1 and (B) GAPDH antibodies on PrEC, PC3, DU145 and LNCaP cells (n=3). (C) Full length western blot using anti-Staufen1 and B-actin antibodies on PC3 cells (n=3). (D) Full length western blot using anti-Staufen1 and (E) B-actin antibodies on LNCaP cells (n=3). (F) Full length western blot using anti-Staufen1 and (G) B-actin antibodies on DU145 cells (n=3). [file 12885_2021_7844_MOESM3_ESM.tif]

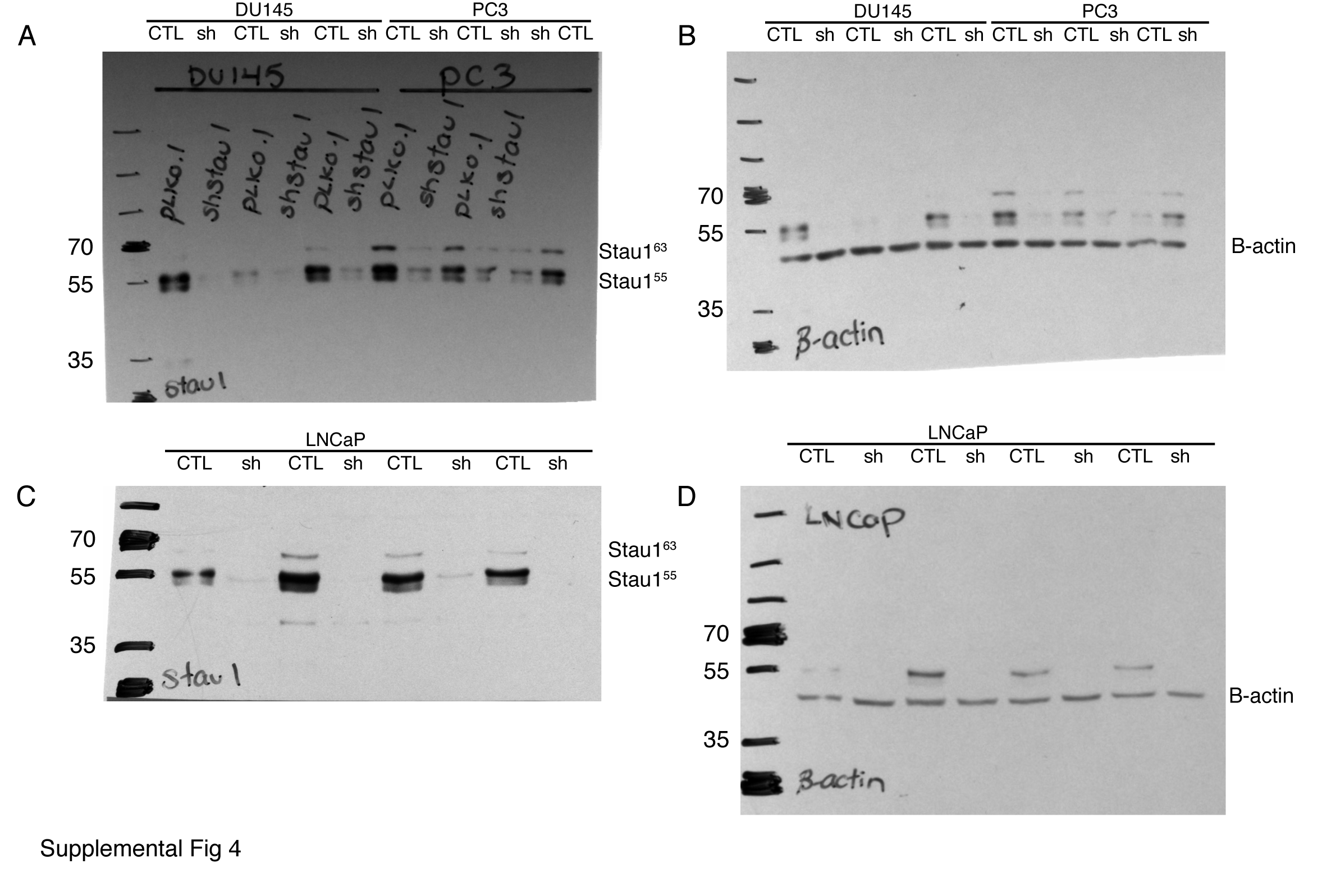

Supplement: Supplementary file 4 — Additional file 4: Supplemental Figure 4. Full length uncropped Western blots for Fig. 2. (A) Full length western blot using anti-Staufen1 and (B) B-actin antibodies on DU145 and PC3 cells (n=3). (C) Full length western blot using anti-Staufen1 and (D) B-actin antibodies on LNCaP cells (n=3). [file 12885_2021_7844_MOESM4_ESM.tif]

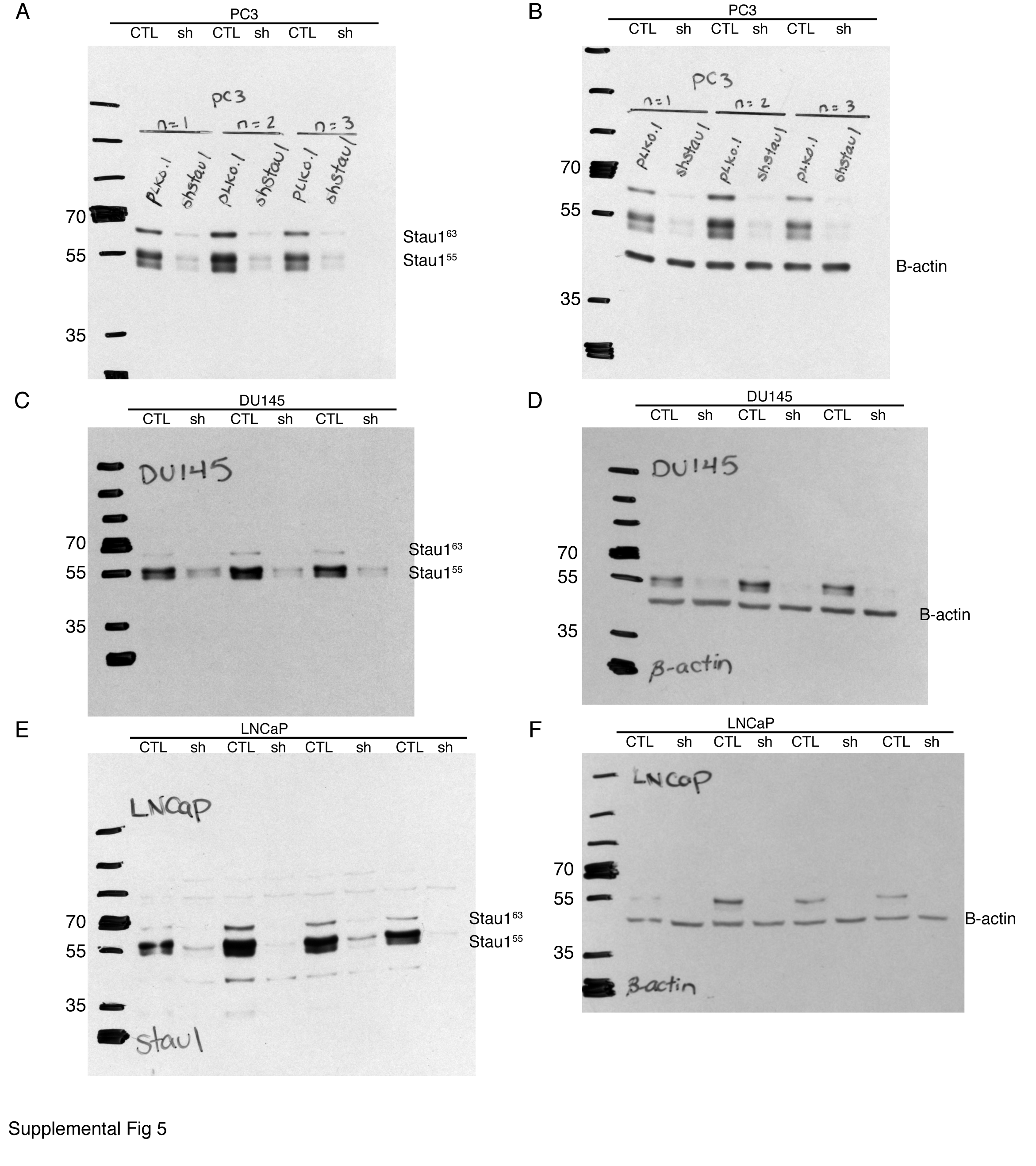

Supplement: Supplementary file 5 — Additional file 5: Supplemental Figure 5. Full length uncropped Western blots for Fig. 3. (A) Full length western blot using anti-Staufen1 and (B) B-actin antibodies on PC3 cells (n=3). (C) Full length western blot using anti-Staufen1 and (D) B-actin antibodies on DU145 cells (n=3). (E) Full length western blot using anti-Staufen1 and (F) B-actin antibodies on LNCaP cells (n=4). [file 12885_2021_7844_MOESM5_ESM.tif]

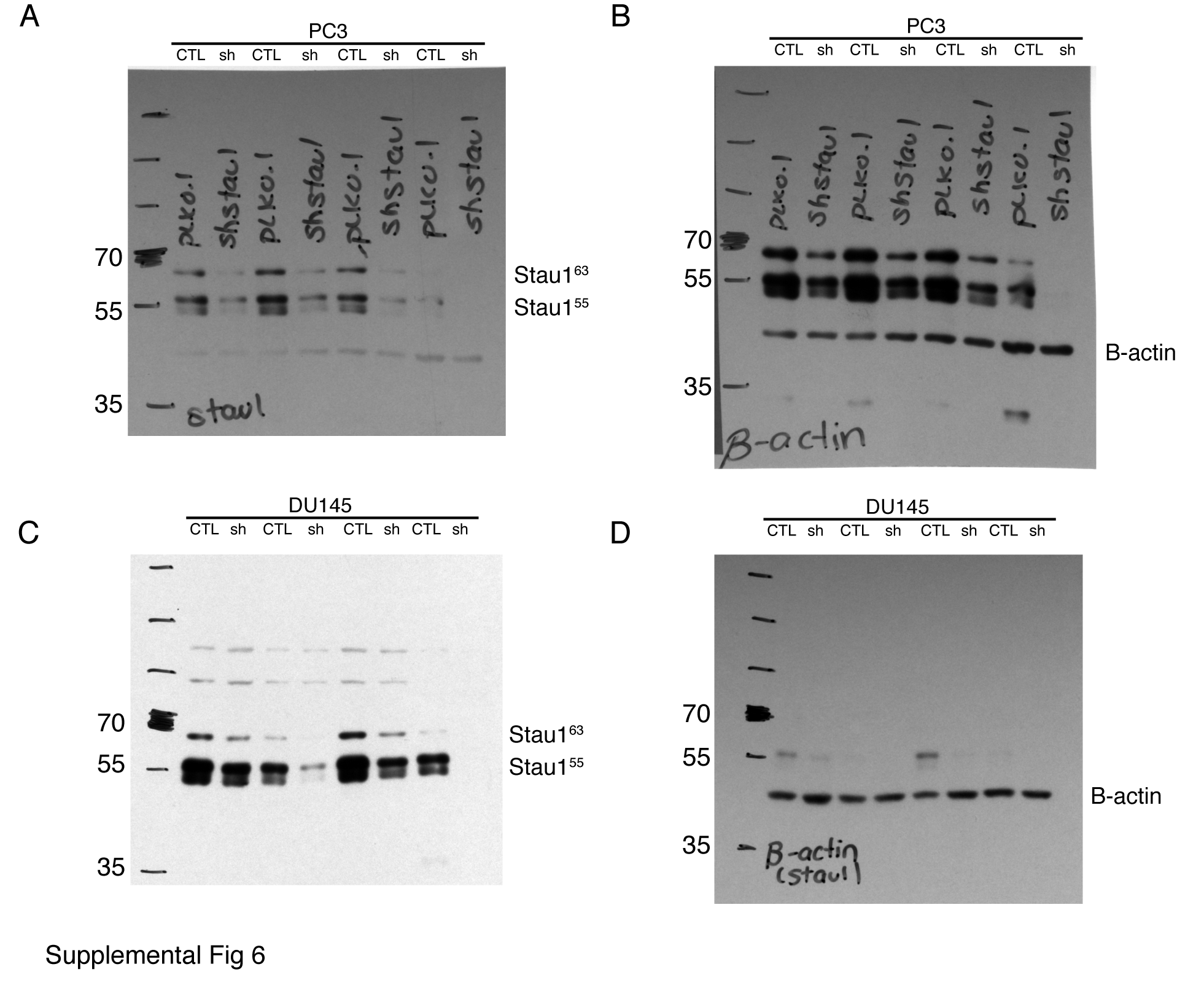

Supplement: Supplementary file 6 — Additional file 6: Supplemental Figure 6. Full length uncropped Western blots for Fig. 4. (A) Full length western blot using anti-Staufen1 and (B) B-actin antibodies on PC3 cells (n=4). (C) Full length western blot using anti-Staufen1 and (D) B-actin antibodies on DU145 cells (n=4). Note: DU145 cell lines used are the same as those presented in Supplemental Figure 6, as the same batch of cells were split and used for both sets of experiments. [file 12885_2021_7844_MOESM6_ESM.tif]

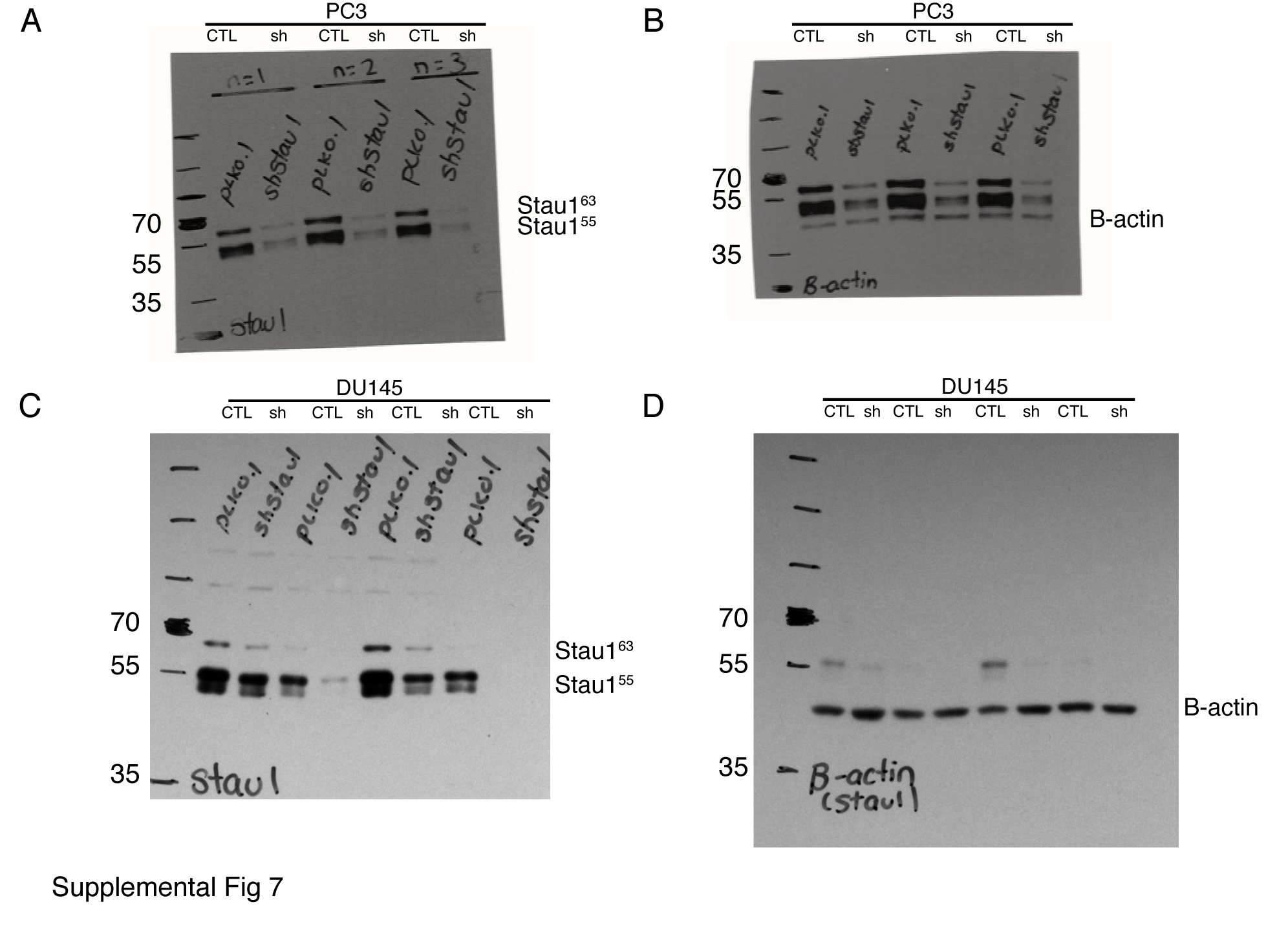

Supplement: Supplementary file 7 — Additional file 7: Supplemental Figure 7. Full length uncropped Western blots for Fig. 5. (A) Full length western blot using anti-Staufen1 and (B) B-actin antibodies on PC3 cells (n=3). (C) Full length western blot using anti-Staufen1 and (D) B-actin antibodies on DU145 cells (n=3). Note: DU145 cell lines used are the same as those presented in Supplemental Figure 5, as the same batch of cells were split and used for both sets of experiments. [file 12885_2021_7844_MOESM7_ESM.tif]

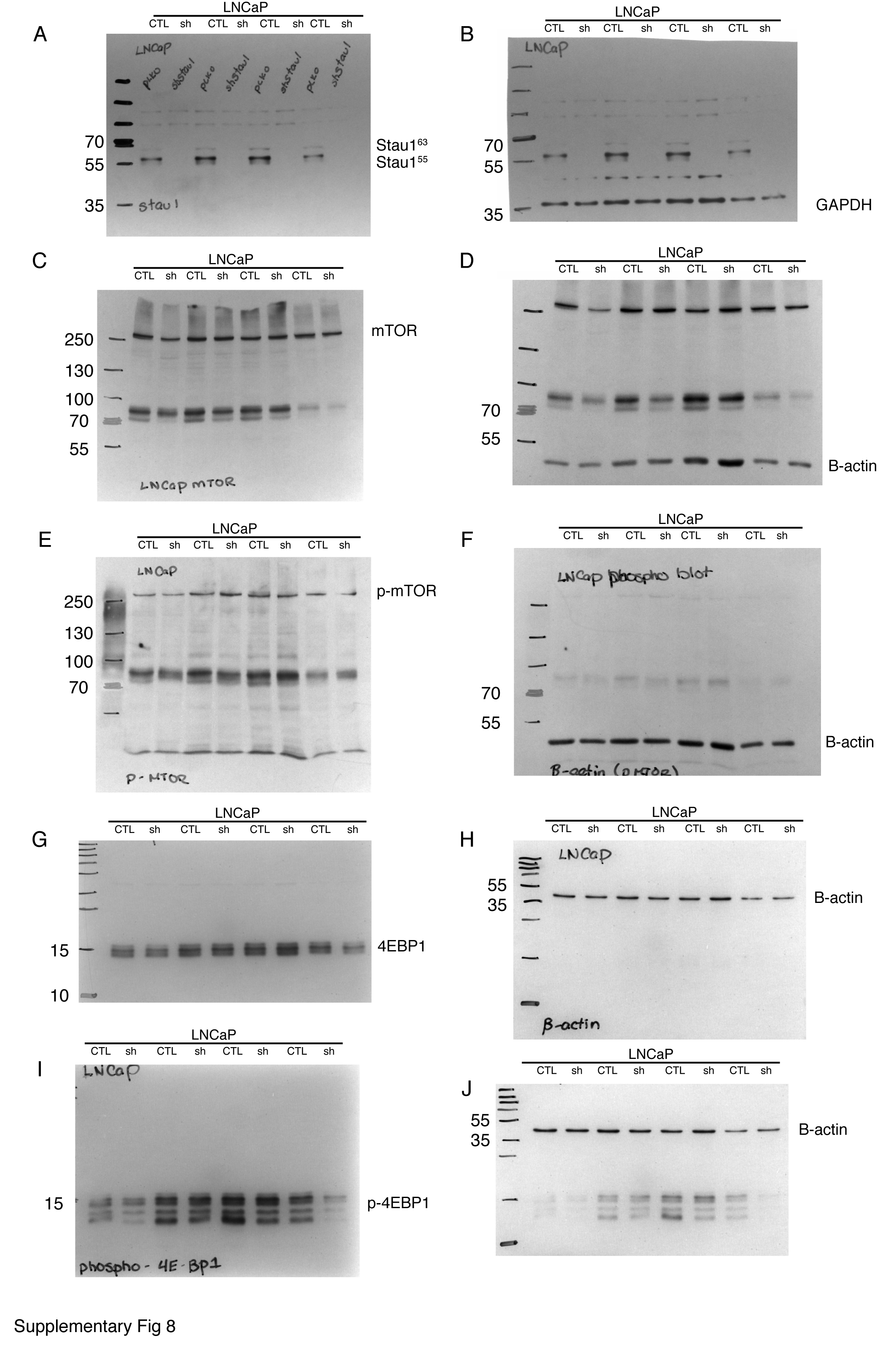

Supplement: Supplementary file 8 — Additional file 8: Supplemental Figure 8. Full length uncropped Western blots for Fig. 6. (A) Full length western blot using anti-Staufen1 and (B) GAPDH antibodies on LNCaP cells (n=4). (C) Full length western blot using anti-mTOR and (D) B-actin antibodies on LNCaP cells (n=4). (E) Full length western blot using anti-phospho-mTOR and (F) B-actin antibodies on LNCaP cells (n=4). (G) Full length western blot using anti-4EBP1 and (H) B-actin antibodies on LNCaP cells (n=4). (I) Full length western blot using anti-phospho-4EBP1 and (J) B-actin antibodies on LNCaP cells (n=4). [file 12885_2021_7844_MOESM8_ESM.tif]

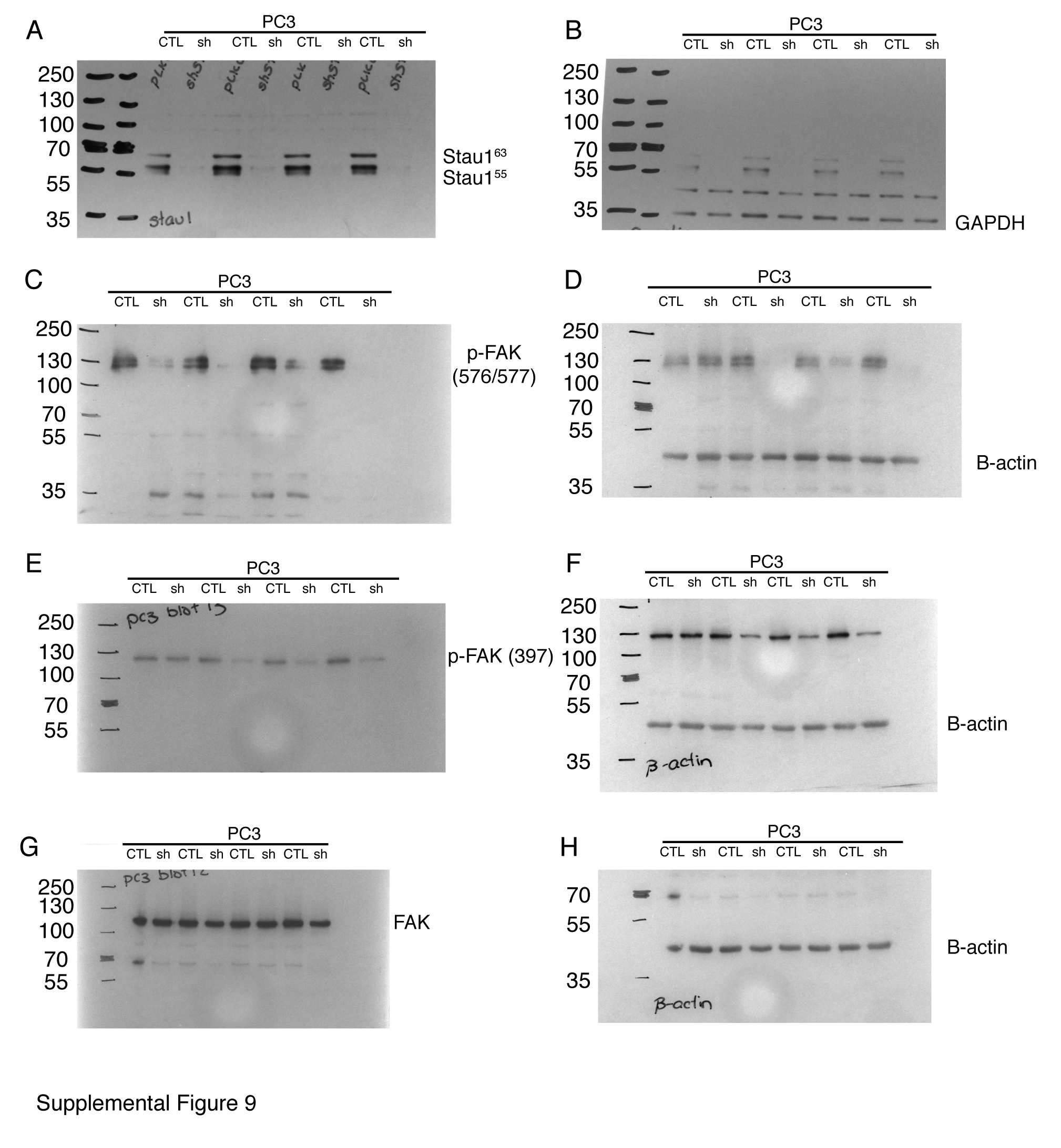

Supplement: Supplementary file 9 — Additional file 9: Supplemental Figure 9. Full length uncropped Western blots for Fig. 6. (A) Full length western blot using anti-Staufen1 and (B) GAPDH antibodies on PC3 cells (n=4). (C) Full length western blot using anti-phospho(576/577)-FAK and (D) B-actin antibodies on PC3 cells (n=4). (E) Full length western blot using anti-phospho(397)-FAK and (F) B-actin antibodies on PC3 cells (n=4). (G) Full length western blot using anti-FAK and (H) B-actin antibodies on PC3 cells (n=4). [file 12885_2021_7844_MOESM9_ESM.tif]
